# Competitive blocking of salivary gland [^18^F]DCFPyL uptake via localized, retrograde ductal injection of non-radioactive DCFPyL: a preclinical study

**DOI:** 10.1186/s13550-021-00803-9

**Published:** 2021-07-21

**Authors:** Jyoti Roy, Blake M. Warner, Falguni Basuli, Xiang Zhang, Changyu Zheng, Corrine Goldsmith, Tim Phelps, Karen Wong, Anita T. Ton, Rick Pieschl, Margaret E. White, Rolf Swenson, John A. Chiorini, Peter L. Choyke, Frank I. Lin

**Affiliations:** 1 Molecular Imaging Program, Center for Cancer Research, National Cancer Institute, NIH, NCI/NIH, Building 10, Room # B3B69F, Bethesda, MD 20892 USA; 2 National Institute of Dental and Craniofacial Research, NIH, Building 10, 1A08, Bethesda, MD 20892 USA; 3 Chemistry and Synthesis Center, National Heart, Lung, and Blood Institute, NIH, Rockville, MD USA; 4 Laboratory of Genitourinary Cancer Pathogenesis, National Cancer Institute, NIH, Bethesda, MD USA

**Keywords:** PSMA, Salivary glands, Radionuclide therapy, Cannulation, Prostate cancer, Xerostomia, Competitive inhibition

## Abstract

**Background:**

PSMA-targeted radionuclide therapy (TRT) is a promising treatment for prostate cancer (PCa), but dose-limiting xerostomia can severely limit its clinical adaptation, especially when using alpha-emitting radionuclides. With [^18^F]DCFPyL as a surrogate for PSMA-TRT, we report a novel method to selectively reduce salivary gland (SG) uptake of systemically administered [^18^F]DCFPyL by immediate prior infusion of non-radioactive standard of [^18^F]DCFPyL (DCFPyL) directly into the SG via retrograde cannulation.

**Methods:**

A dose-finding cohort using athymic nude mice demonstrated proof of principle that SG uptake can be selectively blocked by DCFPyL administered either locally via cannulation (CAN group) or systemically (SYS group). The experiments were repeated in a validation cohort of 22RV1 tumor-bearing mice. Submandibular glands (SMG) of CAN mice were locally blocked with either saline or DCFPyL (dose range: 0.01× to 1000× molar equivalent of the radioactive [18F]DCFPyL dose). The radioactive dose of [18F]DCFPyL was administered systemically 10 min later and the mice euthanized after 1 h for biodistribution studies. Toxicity studies were done at up to 1000× dose.

**Results:**

In the dose-finding cohort, the SYS group showed a dose-dependent 12–40% decrease in both the SMG T/B and the kidney (tumor surrogate). Mild blocking was observed at 0.01× , with maximal blocking reached at 1× with no additional blocking up to 1000× . In the CAN group, blocking at the 0.1× and 1× dose levels resulted in a similar 42–53% decrease, but without the corresponding decrease in kidney uptake as seen in the SYS group. Some evidence of “leakage” of DCFPyL from the salivary gland into the systemic circulation was observed. However, experiments in 22RV1 tumor-bearing mice at the 0.1× and 1× dose levels confirm that, at the appropriate blocking dose, SG uptake of [18F]DCFPyL can be selectively reduced without affecting tumor uptake and with no toxicity.

**Conclusion:**

Our results suggest that direct retrograde instillation of DCFPyL into the SG could predictably and selectively decrease salivary uptake of systemically administered [^18^F]DCFPyL without altering tumor uptake, if given at the appropriate dose. This novel approach is easily translatable to clinical practice and has the potential to mitigate xerostomia, without compromising the therapeutic efficacy of the PSMA-TRT.

**Supplementary Information:**

The online version contains supplementary material available at 10.1186/s13550-021-00803-9.

## Background

**Fig. 1:**
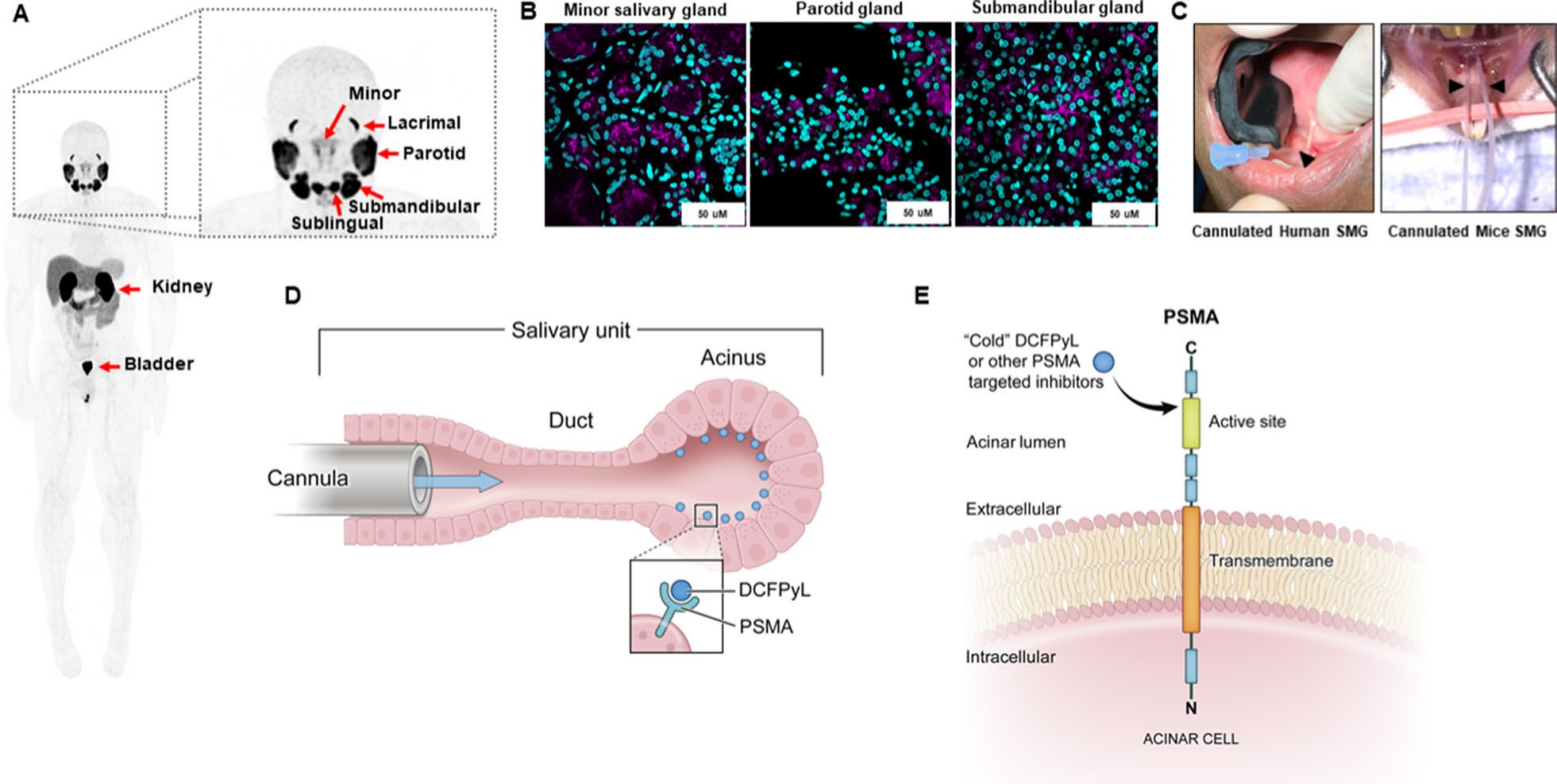
**a** PET/CT scan of a patient administered with [^18^F]DCFPyL demonstrating uptake in the bladder, kidney, salivary (parotid, sublingual, submandibular, and minor/seromucous glands), and lacrimal glands. **b** Immunofluorescence staining of PSMA in human salivary glands (magenta: anti-PSMA staining on the apical lumen of acinar cells; teal: DAPI nuclei staining). **c** Cannulated submandibular salivary glands (SMG) of mouse and human illustrating similarity in delivery of preventive therapy in our model system. Black arrowhead indicates the inserted cannula. **d** and **e** Diagram of the cannulation procedure showing how delivered cold DCFPyL can access and block PSMA sites on the acinar cell surface

Prostate-specific membrane antigen (PSMA) is overexpressed in prostate cancer (PCa) including metastatic castrate-resistant prostate cancer (mCRPC) and has emerged both as a promising imaging marker and as a therapeutic target [1, 2]. While having physiologic uptake limited to areas such as the kidneys, small intestines, lacrimal, and salivary glands (Fig. 1a), the therapeutic efficacy of PSMA-TRT in mCRPC has also been demonstrated clinically in retrospective studies as well as prospective phase 2 [3–7] and the phase 3 VISION trials using the beta-emitting agent ^177^Lu-PSMA-617. Additionally, there is anecdotal clinical evidence that mCRPC patients who have exhausted all other therapies, including ^177^Lu-PSMA-TRT, can still exhibit remarkable response to alpha-emitting PSMA-TRT such as ^225^Ac-PSMA-617[8]. Despite these promising data, xerostomia (dry mouth) remains an important quality-of-life toxicity that can be severe and dose-limiting, especially in patients treated with alpha-emitting PSMA-TRT [1, 9, 10].

The salivary glands (SGs) are comprised of the paired submandibular (SMG), sublingual (SLG), and parotid glands (PRG), as well as numerous minor SGs, all of which contain saliva-producing acinar cells that express PSMA (Fig. 1a, b) [11–13]. These acinar cells are highly sensitive to ionizing radiation and exhibit limited regenerative capacity [14–16]. Severe salivary hypofunction causes difficulty in chewing, swallowing, and speaking and increases the risk of tooth decay and oral infections which reduce quality of life [15, 17, 18]. Damage to salivary acinar cells is directly related to the dose and type of radioactivity (e.g., beta or alpha emitters) administered [1, 15]. Thus, there is a critical need to develop preventive strategies to mitigate this side effect without compromising therapeutic efficacy.

Several strategies, including short-acting anticholinergic drugs, amifostine, local anesthetics, PSMA inhibitors such as 2-PMPA, and botulinum toxin A, have been investigated to mitigate SG toxicity with varying degrees of success [9, 19, 20]. In this investigation, we report on a novel method of salivary protection which we believe can be superior to currently published methods because it can achieve selective salivary uptake reduction without affecting TRT uptake in the PSMA-expressing tumor. Namely, we demonstrate that a localized infusion of DCFPyL, instilled using retrograde cannulation directly into the SG (Fig. 1c, d), can selectively reduce SG uptake of systemically administered [^18^F]DCFPyL via specific binding and competitive inhibition of the PSMA targets in the SG. Salivary gland cannulation is not a technically difficult procedure and is already widely performed in patients clinically as part of a standard sialography (Fig. 1c). For this proof-of-concept study, a fluorine-18-labeled PSMA-targeted PET agent [^18^F]DCFPyL was used as a surrogate for PSMA-TRT in mice [21]. Biodistribution studies were performed to quantify [^18^F]DCFPyL uptake, with or without intraglandular retrograde blocking, in the major SGs, as well as in the tumor and other PSMA-expressing organs such as the kidneys. Toxicology studies were conducted to detect any side effects associated with retrograde injection of DCFPyL into the SGs.

## Methods

All studies were done on protocols approved by the NIH Animal Care and Use Committee.

### Study design

Healthy athymic nu/nu mice (5–6 weeks old, Charles River, 490) were first used in a proof-of-concept and dose-finding cohort to demonstrate that uptake of systemically administered [^18^F]DCFPyL (see Additional file 1: supplementary material for synthesis details) can be blocked at the SG with DCFPyL that was injected either systemically (SYS group) or locally via direct retrograde ductal cannulation of the SG (CAN group). Kidney uptake of [^18^F]DCFPyL was used as a surrogate for tumor uptake in this initial cohort, and the experiment was repeated in 22RV1 tumor-bearing mice after an approximate blocking dose has been determined.

### Generation of mouse tumor xenografts

22RV1 PCa cells were purchased from ATCC and cultured in RPMI 1640 medium supplemented with 10% fetal bovine serum, 1% 2 mM glutamine, 1% penicillin–streptomycin at 37 °C in a 5% CO_2_, and 95% humidified atmosphere. 22RV1 cells [2 × 10^6^ cells per mouse; RPMI 1640/Matrigel (50:50)] were subcutaneously injected near the right shoulder of athymic nude mice (male, 5–6 weeks old, Charles River, 490). Once the tumor volume reached 300–400 mm^3^, mice were used for biodistribution studies. All animal studies and procedures were performed in accordance with NIH IACUC-approved protocols.

### Cannulation procedure of the SG

Mouse submandibular ducts were cannulated as previously described [22]. Briefly, healthy or 22RV1 tumor-bearing male athymic nude mouse was anesthetized by intramuscular injection of a mixture of ketamine chloride (60 mg/kg) and xylazine (5 mg/kg). To reduce saliva secretion and reduce dilution of the injectate, mice were also intramuscularly injected with atropine (0.5 mg/kg). DCFPyL, at doses appropriate for the experiment, or saline, was delivered to the SMG over two minutes by retrograde infusion via a cannula inserted approximately 5 mm into the orifice of the SMG duct (Fig. 1c, d).

### Biodistribution

Injected doses of blocking DCFPyL are expressed as a factor of the molar equivalent of the [^18^F]DCFPyL that was administered systemically, with a dose range of 0.01× to 1000× fold of the systemic dose. At 10 min after administration of DCFPyL, either by IV administration (SYS blocking group) or via retrograde cannulation (CAN blocking group), the systemic (i.v) dose of [^18^F]DCFPyL was administered, and the mice were euthanized after 1 h. Biodistribution studies were performed to determine tissue/blood ratios (T/Bs) of [^18^F]DCFPyL at the time of euthanasia. The effects of the volume of infused DCFPyL were also tested at a standard 50 µL level versus a reduced volume of 25 µL.

### Systemic studies (SYS blocking group)

**Fig. 2:**
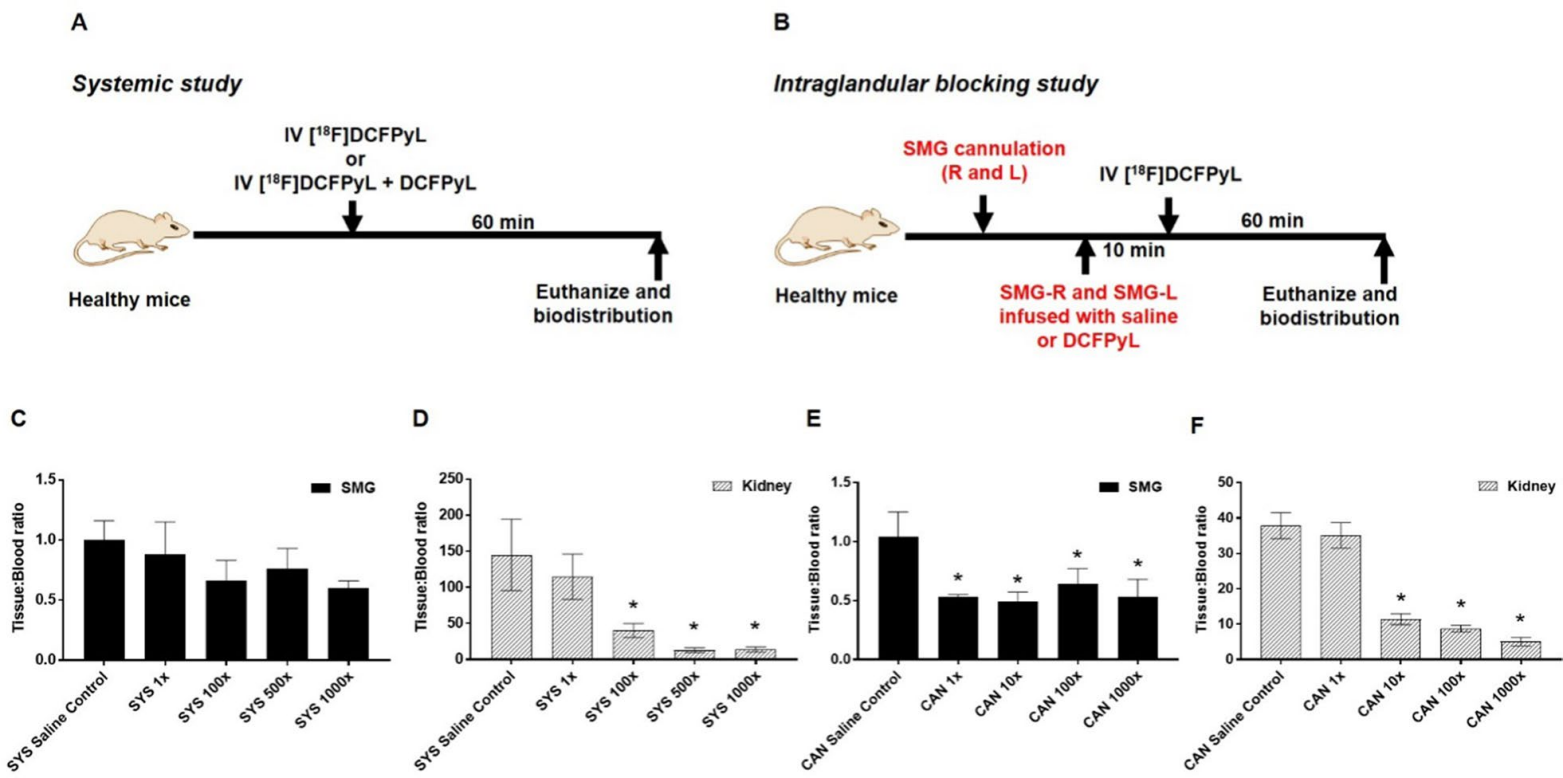
Experimental design for systemic study (SYS group) and intraglandular blocking study (CAN group) for the dose-finding in non-tumor-bearing cohort (**a**, **b**). The tissue/blood ratios of [^18^F]DCFPyL in submandibular salivary glands (SMG) and kidney at 1 h post-injection are shown for the SYS group (panels **c**, **d**) and for the CAN group (panels **e**, **f**), respectively. Mice were injected either with 50 µl of saline control or with DCFPyL at 1× to 1000× molar equivalent of the systemically injected [^18^F]DCFPyL dose. Each value in the graphs represents mean tissue/blood ratios ± SD, *n* = 5–6 for each group, with statistically significant results (*P* < 0.05) indicated by asterisk. The CAN 1 × group shows the expected local blocking at the SMG and lack of blocking at the kidneys (tumor surrogate). However, unexpected blocking of the kidneys is seen in the CAN group at the 10–1000× level, perhaps due to “leakage” of DCFPyL from intraglandular administration into the systemic circulation

Healthy male mice (5–6 weeks old, athymic nude, Charles River, 490) were randomly divided into saline control or blocking groups. Mice in the saline control group (SYS saline control) were intravenously administered with 100 µCi of [^18^F]DCFPyL, whereas mice in blocking groups were intravenously administered with the same dose of radioactivity along with 0.1-fold, onefold, 100-fold, 500-fold, or 1000-fold excess of DCFPyL (SYS 0.1× , SYS 1× , SYS 100× , SYS 500× , and SYS 1000× ; Fig. 2a). For the validation cohort, 22RV1 tumor-bearing mice were either injected with [^18^F]DCFPyL (100 µCi, i.v) alone or in the presence of 0.1 or onefold excess of DCFPyL (Fig. 4a). Mice were euthanized (CO_2_ asphyxia) 1 h post-injection of [^18^F]DCFPyL, and biodistribution was performed. Radioactivity (counts per minute, CPM) associated with the tissue was determined using a gamma counter (PerkinElmer 2480 Wizard3; counting efficiency 52.4%, 400–1200 keV). The radioactive content (CPM) of all the tissue samples was background corrected and decay corrected to the start time of sample counting. Percentage injected dose per gram (%ID/g) and tissue/blood ratio were determined as follows:

1. $$\% {\text{ID}}/{\text{g }} = {\text{ }}\left\{ {\left[ {{\text{CPM}}_{{{\text{tissue}}}} /{\text{CPM}}_{{{\text{total injected dose}}}} } \right]/\left[ {{\text{tissue weight }}\left( {\text{g}} \right)} \right]} \right\} \times {\text{1}}00$$
2. $$\% {\text{ID}}/{\text{g}}_{{{\text{normalized to a 2}}0{\text{ g mouse}}}} = {\text{ }}\left( {\% {\text{ID}}/{\text{g}}} \right) \times \left[ {\left( {{\text{body weight of mice}}} \right)/\left( {{\text{2}}0{\text{ g}}} \right)} \right]$$
3. $${\text{Tissue}}:{\text{Blood Ratio}} = \frac{{{\text{Tissue }}\% {\text{ID}}/{\text{g}}_{{{\text{normalized to a 2}}0{\text{g mice}}}} }}{{{\text{Blood }}\% {\text{ID}}/{\text{g}}_{{{\text{normalized to a 2}}0{\text{g mice}}}} }}.$$

### Intraglandular blocking studies (CAN blocking group)

**Fig. 3:**
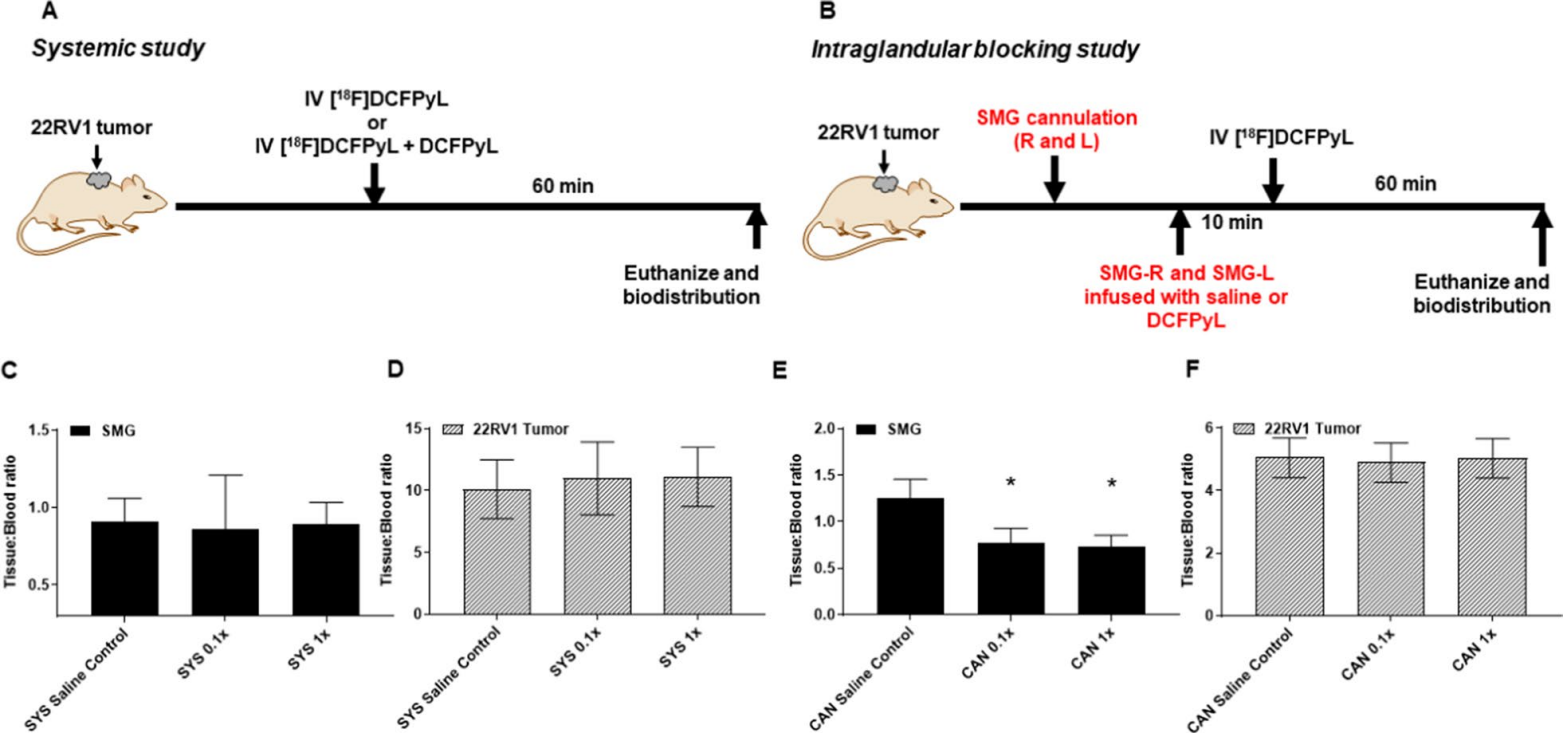
Experimental design for systemic study (SYS group) and intraglandular blocking study (CAN group) in the validation 22RV1 tumor-bearing cohort (**a**, **b**). The tissue/blood ratios of [^18^F]DCFPyL in submandibular salivary glands (SMG) and the tumor at 1 h post-injection are shown for the SYS group (panels **c**, **d**) and for the CAN group (panels **e**, **f**), respectively. Mice were injected either with 50 µl of saline control or with “cold” DCFPyL at 0.1 × or 1 × molar equivalent of the systemically injected “hot” [^18^F]DCFPyL dose. Each value in the graphs represents mean tissue/blood ratios ± SD, *n* = 5–7 for each group, with statistically significant results (*P* < 0.05) indicated by asterisk. Mice in the CAN 0.1 × and 1 × groups demonstrate statistically significant local blocking at the SMG with no reduction in [^18^F]DCFPyL uptake at the tumor

Submandibular salivary glands (SMG) of non-tumor-bearing healthy male mice (5–6 weeks old, athymic nude, Charles River, 490) were cannulated as previously described (Fig. 1c) [22]. Mice were randomized into saline control and blocking groups. Right and/or left SMG glands (SMG-R, SMG-L) of mice in the control group were infused retrograde with either 25 or 50 µl sterile saline (CAN saline control). SMG-R and/or SMG-L glands of mice in the blocking group were infused retrograde with 25 or 50 µl of either 0.01-fold, 0.1-fold, onefold, tenfold, 100-fold, or 1000-fold excess of unlabeled DCFPyL (CAN 0.01× , CAN 0.1× , CAN 1× , CAN 10× , CAN 100× , and CAN 1000× ; Figs. 2d and 3a). For the validation cohort, right and left SMGs of 22RV1 tumor-bearing mice were cannulated and the animals were randomized into control and blocking groups. Cannulated SMG-R and SMG-L of control mice were infused with 50 µl of sterile saline (CAN saline control), whereas both the glands of mice in the blocking group were infused with 50 µl of 0.1 or onefold excess of DCFPyL (CAN 0.1× , CAN 1× ; Fig. 5a). Ten min after retrograde infusion of saline or DCFPyL in the SMG glands, mice in all groups were injected (i.v) with 100 µCi of [^18^F]DCFPyL. One hour post-injection of the radioactive compound, mice were euthanized (CO_2_ anaphylaxis) and biodistribution was performed. The amount of radioactivity (CPM) associated with various organs/tissues was determined using a gamma counter, and %ID/g of tissue and T/B ratios were calculated using the formulas described earlier.

### Toxicity evaluation

Short- and long-term toxicity of the cannulation procedure was examined at 1- and 2-month time points and is detailed in supplementary material.

### Data analysis

Data were analyzed using GraphPad Prism7. The statistically significant difference (*P* < 0.05) between the groups was determined using the two-sample Student’s t-test assuming unequal variances.

## Results

### Biodistribution studies: dose-finding cohort

Initial proof-of-concept and dose-finding experiments were done using non-tumor-bearing mice to see whether PSMA-mediated SG of [^18^F]DCFPyL can be blocked at all by DCFPyL given either systemically (SYS study group) or locally via retrograde cannulation (CAN study group). Tissue-to-blood (T/B) ratios were calculated to account for the variations observed in the input function (blood retention) of [^18^F]DCFPyL in the various groups. Biodistribution of [^18^F]DCFPyL in non-tumor-bearing mice demonstrated that the highest uptake of radioactivity was in the kidneys (Additional file 1: SI Figure 2), consistent with prior reports [23].

For mice in the SYS blocking groups, it was found that systemic administration of DCFPyL reduced the SG T/B ratios (Fig. 2c) compared to mice that were given a systemic saline control, although not at a statistically significant level. Mice in SYS 1×, SYS 100×, SYS 500×, and SYS 1000× groups exhibited a 12%, 34%, 24%, and 40% decrease in T/B ratios of the SMGs, respectively. Since PSMA is known to be expressed in mouse kidney, renal uptake of [^18^F]DCFPyL was employed as a surrogate for tumor uptake in this non-tumor-bearing cohort. This surrogacy allowed us to perform a preliminary evaluation of whether the blocking dose of DCFPyL can indeed selectively reduce the accumulation of [^18^F]DCFPyL in the SG, without affecting the kidney (tumor surrogate) [^18^F]DCFPyL uptake. Since the DCFPyL was given systemically in these SYS blocking groups, it is expected that renal [^18^F]DCFPyL would also be blocked. Indeed, a statistically significant 73–91% reduction in the renal T/B of [^18^F]DCFPyL was observed in SYS 100×, SYS 500×, and SYS 1000× groups, although only a nonsignificant 21% decrease was noted in the SYS 1× group (Fig. 2d).

Compared to the SYS blocking groups, biodistribution of [^18^F]DCFPyL in the CAN study groups showed overall higher radioactivity in all organs (except liver; Additional file 1: SI Figures 2 and 3), which is likely an artifact related to anesthesia used during the cannulation procedure and the resultant slower elimination of unbound [^18^F]DCFPyL. Mice in the CAN groups demonstrated more efficient blocking of the SGs compared to the corresponding SYS group with an observed reduction of 42–53% in the SMG T/B that reached statistical significance (Fig. 2e). Interestingly, although DCFPyL was instilled only into the SMG, a reduction in the renal T/B of [^18^F]DCFPyL was also observed in the CAN group in a dose-dependent fashion, perhaps due to “leakage” of DCFPyL from intraglandular administration into the systemic circulation. Whereas 70–86% of [^18^F]DCFPyL uptake in the kidney was blocked in the CAN 10×, CAN 100×, and CAN 1000× groups, only a 7.4% reduction was observed in the CAN 1× group (Fig. 2f).

**Fig. 4:**
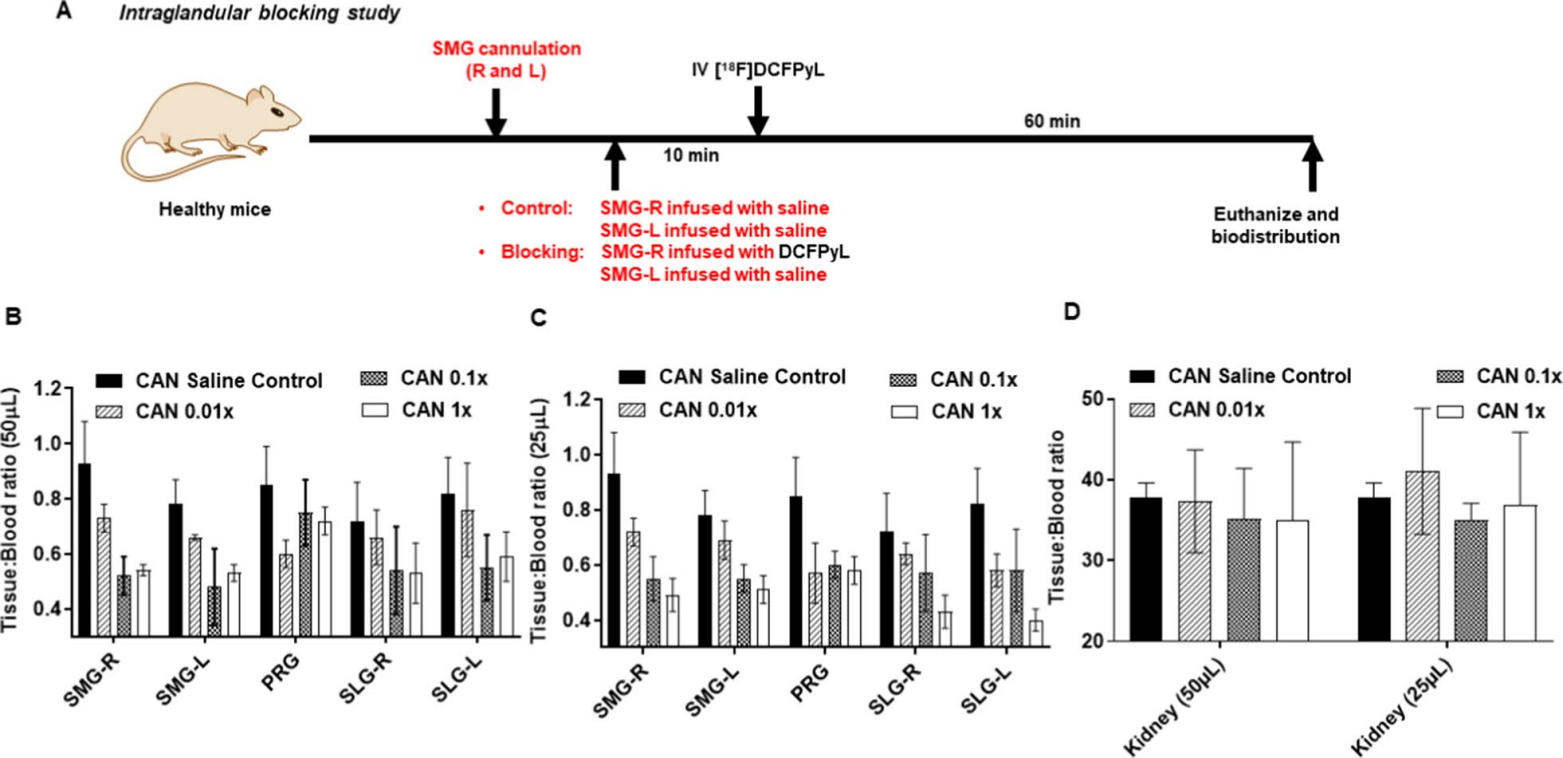
**a** Experiment design for intraglandular blocking study. **b**, **c** Tissue/blood ratios of [^18^F]DCFPyL in salivary glands (submandibular: SMG, sublingual: SLG, parotid: PRG, right: R, left: L; **b** and kidneys (**c**) at 1 h after injection. SMG glands were cannulated infused either with saline (CAN saline control) or with 25 µl or 50 µl of DCFPyL at 0.01, 0.1, 1 × molar excess of the systemically injected “hot” [^18^F]DCFPyL dose. Ten minutes after SMG infusion, mice were injected with [^18^F]DCFPyL. Each value in the graph represents mean tissue/blood ratios ± SD, *n* = 4–6 for each group

Since it appeared that the selectivity of SG PSMA blocking may be related to the dose of blocking material used in the CAN group, further experiments were performed using lower concentrations of DCFPyL. As the degree of blocking appeared to have plateaued at 1×, repeat experiments were done at 0.01×, 0.1× and 1× doses, which yielded reductions in the SMG T/B ratio of 21.5%, 44.1%, and 41.9%, respectively. At the 0.01× and 0.1× dose levels, no significant reduction (< 2% decrease) in kidney T/B ratios was observed (Fig. 4d).

### Validation tumor-bearing cohort

Using data from the dose-finding, non-tumor-bearing cohort, the optimal dose of DCFPyL to achieve selective SG blocking was determined to be between 0.1× and 1× molar equivalent of the radiolabeled [^18^F]DCFPyL dose that was given systemically. Therefore, the biodistribution experiments were repeated in 22RV1 tumor-bearing mice at these two dose levels, following the same study design of giving the DCFPyL blocking agent both systemically (SYS group) and locally via direct cannulation of the SG (CAN group).

As was expected, only minimal decrease in the SMG T/B ratio was observed in the SYS 0.1× and SYS 1× groups (Fig. 3c) when compared with control mice. Also as expected, no observable decrease in the tumor T/B ratios was noted in these dose groups (Fig. 3d). On the other hand, infusion of 0.1 ×and 1× of DCFPyL directly into the SG in the CAN group did reduce the SMG T/B by approximately 40% compared to control (Fig. 3e), which validates the previous results seen in the non-tumor-bearing mice. More importantly, no significant decrease in the tumor T/B ratios was noted (Fig. 3f), which is indicative of selective [^18^F]DCFPyL blocking at the salivary gland, but not at the PSMA-positive tumor.

### Effects on contralateral and other non-cannulated salivary glands

Although only the right SMG was injected with the DFCPyL, reductions in the [^18^F]DCFPyL T/B ratios were also observed in the contralateral SMG as well as the other non-cannulated SG. For instance, in the SYS group of the dose-finding cohort, reduced T/B ratio was also observed in the PRG (SYS 1 × : 4.4%, SYS 100 × : 20%, SYS 500 × : 17%, 1000 × : 26%) and the SLG (SYS 1 × : 23%, SYS 100 × : 23%, SYS 500 × : 53%, SYS 1000 × : 50%; Additional file 1: SI Figure 4A). Similarly, mice in the CAN 1 × group of the dose-finding cohort exhibited 28.7% (PRG) and 28.2% (SLG) reduced T/B ratios compared to CAN saline control (Additional file 1: SI Figure 4B). Mice in the CAN 10 × , 100 × , and 1000 × groups showed a 37–39% reduction in PRG T/B and a 30–50% reduction in SLG T/B (Additional file 1: SI Figure 4B). In the 22RV1 tumor-bearing mice, even though only the right SMG was instilled with DCFPyL, decreased T/B was also detected in the left SMG (CAN 0.1 × :32%; CAN 1 × :29%; Additional file 1: SI Figure 5).

### Effects of volume reduction

Experiments were also performed to determine whether reducing the volume of infusion from 50 to 25 µl can prevent or reduce the observed blocking seen at non-cannulated SGs such as the contralateral SMG (Fig. 4). Repeat cannulation and biodistribution experiments show that even with the smaller infusion volume of 25 µl, the degree of blocking between the cannulated right SMG and the non-cannulated left SMG remains similar. The T/B ratios of the right SMG at the 0.01 × , 0.1 × , and 1 × levels are 0.72, 0.55, and 0.49, respectively, whereas the T/B ratios of the control left SMG at these same dose levels are 0.69, 0.55, and 0.51, respectively (Fig. 4b).

### Toxicity evaluation

**Fig. 5:**
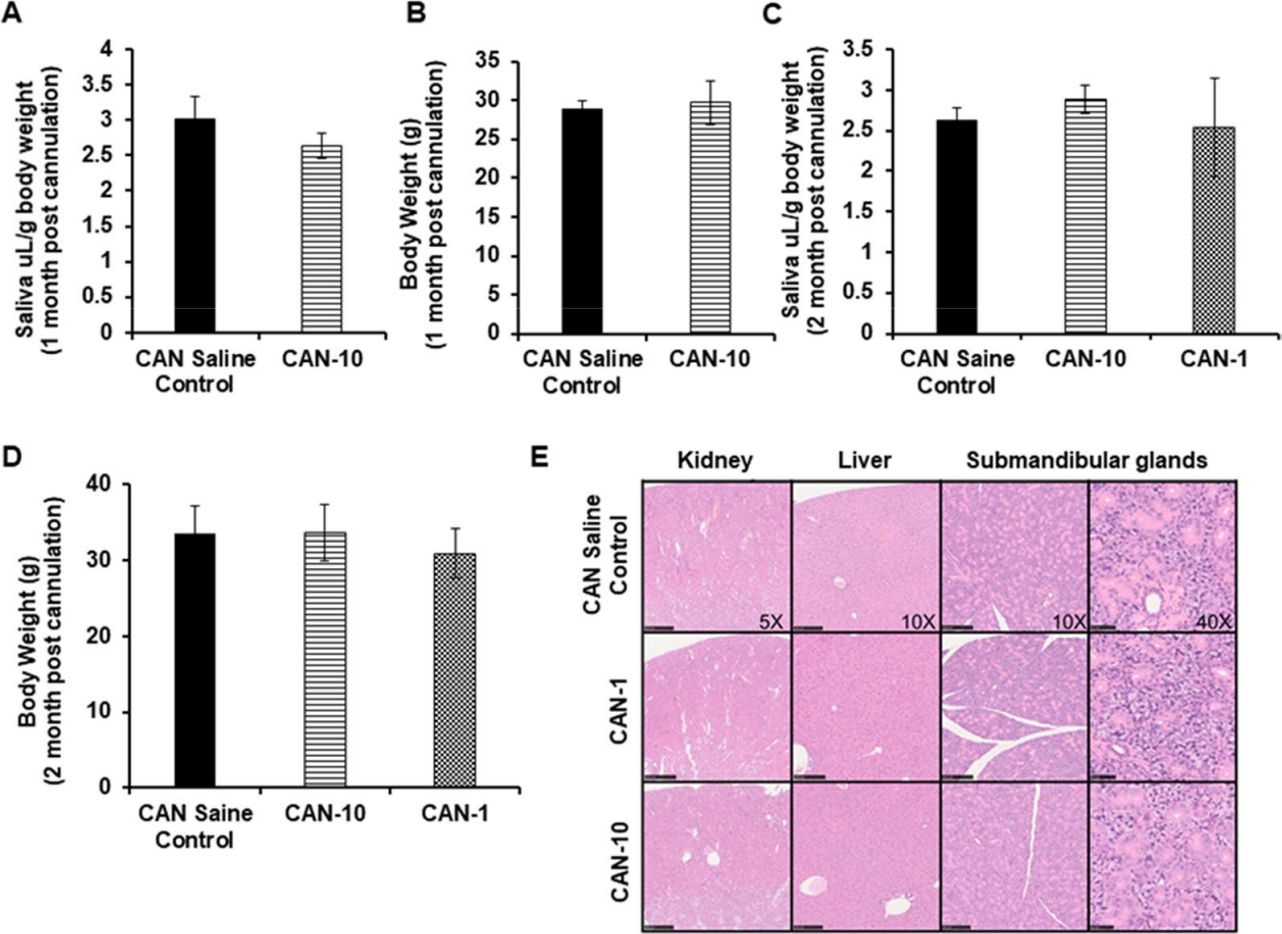
Effect of instillation of 10 nmoles (CAN-10) and 1 nmoles (CAN-1) of DCFPyL in submandibular glands via cannulation at different times (1 or 2 months) on saliva secretion (**a**, **c**) and body weight (**b**, **d**). **a** and **c** Each bar represents mean saliva volume (µL) normalized to body weight (g) ± SD. **b** and **d** Each bar represents mean body weight (g) ± SD, *n* = 4–6 each group. **e**: There were no microscopic differences (H&E) between the kidney, liver, and submandibular gland of various groups

Intraglandular administration of DCFPyL yielded no discernible adverse effects across the evaluated parameters. Saliva flow rate is a measure of glandular activity, and normal flow rate is evidence of a healthy and functional SG. There were no differences in saliva flow rates between CAN saline control and blocking groups (CAN-10 and CAN-1) at one-month (Fig. 5a) or two-month (Fig. 5c) time points. Compared to the CAN saline control group, there were no differences in animal weights in mice in CAN-10 and CAN-1 groups, indicating no cumulative toxicity (Fig. 5b, d). No abnormal changes in amylase levels and liver or kidney functional biomarkers were observed among the groups (Additional file 1: SI Figure 6). Lastly, there were no microscopic differences in the kidneys, livers, or SMGs of CAN-10 or CAN-1 compared to the organs from the CAN saline control group (Fig. 5e).

## Discussion

Although data with PSMA-TRT have thus far been very promising in the treatment of prostate cancer, SG damage leading to xerostomia could potentially limit its clinical adaptation, especially when PSMA-TRT with alpha particles is used. In order to fully realize the potential of PSMA-TRT, there is an urgent need to develop strategies to limit SG toxicity without compromising its therapeutic efficacy [9, 15]. Herein, we investigated a novel strategy that has the potential to safely and effectively mitigate SG damage. We tested the hypothesis that a localized infusion of unlabeled (“cold”) PSMA ligand directly into the SG can selectively reduce SG uptake of radiolabeled “hot” PSMA agents via competitive inhibition of the receptor target, without affecting uptake at the PSMA-positive tumor.

In our proof-of-concept and dose-finding cohort, we were able to demonstrate that, not surprisingly, uptake at PSMA-positive targets can be blocked by administering DCFPyL as either a systemic (SYS group) or a localized (CAN group) infusion. We also expected that in the SYS group, all PSMA-positive targets in the body including the kidney (tumor surrogate) would be blocked, whereas in the CAN group, only the SG would be selectively blocked. This is in fact what we observed, and the findings were repeated and confirmed in a 22RV1 tumor-bearing cohort. There were, however, two surprising results from our experiments.

First, from our dose-finding cohort, we showed that DCFPyL was able to block uptake of [^18^F]DCFPyL at the SG in a dose-dependent fashion, testing from a dose range of 0.01 × to 1000 × molar equivalent of the systemically injected hot dose. We found that DCFPyL administered locally via cannulation was more effective at blocking the SG when compared to an equivalent blocking dose administered systemically. For instance, a 1 × blocking dose decreased uptake at the SMG by 12% when given systemically but decreased uptake by 49% when infused directly into the SG. This is likely because the concentration of the blocking DCFPyL seen at the SG is greatly diluted by the systemic blood volume of distribution when administered systemically. We also found that when looking only at the CAN group, a blocking dose of 0.01 × sub-optimally blocked uptake with a 21.5% reduction, but the blocking efficiency quickly increased with dose up to approximately 41–49% at the 1 × dose. Despite this apparent dose dependency, the blocking percentage appears to plateau at the 1 × dose, and we were unable to elicit higher levels of blocking even with increases in dose up to 1000 × . One possible explanation is that there could be both PSMA receptor-specific and non-specific components (such as passive diffusion) to the observed salivary [^18^F]DCFPyL accumulation, and the DCFPyL was only able to block the receptor-specific component. Our findings seem to agree with prior published data using pig SG autoradiography of [^177^Lu]Lu-PSMA-617 demonstrating both PSMA-specific and non-specific uptake in the SG [24].

The second surprising finding from our experiments was that in the CAN group, non-cannulated sites such as the contralateral SG and distant sites such as the kidney and the flank tumor also demonstrated blocking of PSMA uptake. As DCFPyL was administered only to the SG in the CAN group, there must be some kind of “leakage” of the blocking agent from inside the SG into the surrounding area or into the systemic circulation to account for this finding. Furthermore, we found that the degree of blocking in these non-cannulated sites appears to be related to proximity both to the cannulated SG and to the dose of injected blocking agent. These results have been observed with other studies examining cannulation of the SGs [25].

As demonstrated in Fig. 4, even though only the right SMG was instilled with DCFPyL, other regional SGs such as the right SLG and the PRG also demonstrated blocking. For instance, at the 1 × dose level, mice in the CAN group demonstrated a 41.9% blocking in the cannulated right SMG, but the un-cannulated PRG and SLG also showed blocking of 28.7% and 28.2%, respectively, even though no DCFPyL was administered directly to those glands. Another piece of this puzzle is the finding that while intraglandular administration of DCFPyL at the 1 × dose level did not result in blocking of distant sites such as the kidney and the tumor, higher dose levels at 10–1000 × did (Fig. 2f).

Putting these findings together, one can only speculate at the mechanism of this “leakage.” One possible explanation, as stated earlier, could be a non-specific component of PSMA accumulation in the SG due to processes such as passive diffusion. As part of the cannulation and infusion procedure (where the infused volume is held in place in the SG by a locked syringe), it is possible that the increased hydrostatic pressure generated could facilitate paracellular diffusion of DCFPyL out of the cannulated gland and into the surrounding soft tissue, and from there, into regional SGs via the lymphatic vasculature. Although our experiment focused on decreasing the infused volume from 50 to 25 µl did not seem to reduce the blocking seen in non-cannulated glands, an even lower volume would be needed to eliminate this effect (which we cannot test due to technical limitations of the procedure in mice). Regardless of the mechanism of this regional blocking, it is clear that the leaked DCFPyL does enter the systemic circulation at some point since this is the only plausible explanation for why distant sites such as the kidney and tumor are also blocked. Lymphatic collection of leaked DCFPyL which then gets dumped backed into the systemic circulation could be one possible mechanism.

Despite this observed leaking phenomenon, our experiments demonstrated that selective blocking of the SG is still achievable as long as an appropriate blocking dose of the DCFPyL is used intraglandularly. For instance, in the 22RV1 tumor-bearing mice, we demonstrated that at the 0.1 × and 1 × dose levels, the SMG-R can be blocked by approximately 40% and at a statistically significant level without affecting the uptake of [^18^F]DCFPyL at the PSMA-positive tumor (Fig. 3e, f). This selective SG blocking effect is not seen when the same dose levels were injected systemically (Fig. 3c, d). While there could be many potential explanations, we conjecture that this is due to a first-pass effect, where the SGs are exposed to much higher concentrations of the blocking DCFPyL compared to the tumor because of the localized injection directly into the SG. With higher injected dose (at 10 × and higher in our experiment), the amount of DCFPyL entering the systemic circulation likely becomes high enough that the benefit of the first pass effect is lost and selectivity of blocking at the SG is no longer seen.

While our experiments are performed in mice, we believe that this approach is highly translatable to humans as salivary duct cannulation is already routinely done clinically for procedures such as sialography. Since clinical sialography is done with iodinated contrast that can be visualized and that contrast extravasation into the surrounding soft tissue is not routinely seen in sialography, it is possible that the “leakage” phenomenon observed in our mice experiment will not become an issue when performed in humans. However, human clinical trials using this approach are needed to test this hypothesis.

Compared to other published methods of SG damage mitigation, we believe our method can produce superior and more consistent blocking results. For instance, sialoendoscopy has been evaluated in patients undergoing ^225^Ac-PSMA-617 therapy to prevent xerostomia, and in one study, the SMG and PRG were cannulated and irrigated with sterile saline and prednisolone injections [26]. Although some initial improvement was observed, it was unclear whether the effect was due to saline irrigation or administration of the steroids. Encouraging results were also observed in a preclinical and clinical trial evaluating botulinum toxin A; however, further investigation in more patients is needed to determine the dosing and timing of botulinum injection and the possibility of long-term SG damage from the botulinum injection itself should be carefully considered [27, 28]. In a preclinical PCa tumor model, monosodium glutamate (MSG) showed reduced uptake of ^68^Ga-PSMA-11 in SGs and kidney without affecting tumor uptake [29]. But further investigation is needed to determine the amount of MSG needed to effectively block the off-target uptake of ^68^Ga-PSMA-11. Furthermore, a recently published clinical study of 16 patients using both orally ingested and topical MSG demonstrated that while SG uptake of ^68^Ga-PSMA-11 can be reduced with this intervention, tumor uptake of ^68^Ga-PSMA-11 also declined which makes this approach unlikely to be clinically useful in the setting of TRT [30]. Administration of precursor of PSMA inhibitor 2-(phosphonomethyl) pentanedioic acid (2-PMPA) before ^177^Lu-PSMA-617 site-specifically blocked the uptake of PSMA-TRT in the SGs and kidneys without affecting the accumulation of the agents in the tumor, but this approach also needs further studies to validate its effectiveness [31].

Lastly, it is noted that Kalidindi et al. recently published a preclinical investigation suggesting that systemic administration of cold PSMA-11 ligand can decrease SG uptake of ^177^Lu-PSMA-617 in mice with PC3-PIP xenografts without significantly affecting tumoral uptake [32]. The authors suggest that the SG might be blocked more effectively than the tumor due to earlier SG receptor saturation/blocking compared to the tumor, which is a valid hypothesis. However, if this theory is true, then one would suspect that the amount of SG blocking actually achieved in patients will likely be highly unreliable and unreproducible, as the amount of cold PSMA ligand that the SG is exposed to will be heavily influenced by factors such as differences in each individual patient’s tumor load, amount of physiologic uptake in organs such as the liver/kidneys, and other pharmacokinetic variables. Similarly, the amount of blocking at the PSMA + tumor will likely be highly variable as well. This point is supported by our set of experiments in the SYS blocking group, where we demonstrated that differing doses of systemically administered DCFPyL have differing blocking effects on the tumor, anywhere from 21% to as high as 90% decrease in [^18^F]DCFPyL uptake. Such contrasting results suggest the need for further investigations, but the benefit of the cannulation blocking approach lies in the ability to consistently deliver much higher concentration of blocking DCFPyL to the SG via the first pass effect, which leads to a more selective and effective SG blocking compared to systemic blocking.

While the results of these experiments are promising, potential limitations to the generalizability of these data are noted. For instance, while blocking is demonstrated in the mice model, there could be physiologic differences between mice and human salivary glands which make further investigations in clinical trials essential prior to routine clinical use in patients. Furthermore, salivary blocking in this study is demonstrated using the imaging agent [^18^F]DCFPyL, which suggests but does not guarantee that blocking would be observed when a similar but different therapeutic ligand such as [^177^Lu]PSMA-617 is used. Further investigations would be needed to confirm these results.

## Conclusions

Our results suggest that direct retrograde infusion of DCFPyL into the SG could selectively decrease SG uptake of systemically administered [^18^F]DCFPyL without altering tumor uptake, if given at the appropriate dose. This novel approach is easily translatable to clinical practice and has the potential to mitigate xerostomia, without compromising the therapeutic efficacy of the PSMA-TRT. However, future studies including validation in a clinical trial are needed to further confirm these findings.

## Supplementary Information

**Additional file 1**. SI Figure 1–SI Figure 6.

## Data Availability

The datasets used and/or analyzed during the current study are available from the corresponding author on reasonable request.
