# Supplementary material for: Competitive blocking of salivary gland [18F]DCFPyL uptake via localized, retrograde ductal injection of non-radioactive DCFPyL: a preclinical study
Source: EJNMMI Res. 2021 Jul 21;11:66. doi: 10.1186/s13550-021-00803-9 (PMC8295433; doi:10.1186/s13550-021-00803-9)
Supplement: Supplementary file 1 — Additional file 1. SI Figure 1–SI Figure 6. [file 13550_2021_803_MOESM1_ESM.docx]

**SUPPLEMENTARY INFORMATION**

**METHODS**

**Synthesis of [^18^F]DCFPyL**

[^18^F]DCFPyL was synthesized according to a published procedure in the literature with 32-43% radiochemical yield and >98% purity (SI Figure 1). The molar activity was 1200-2600 Ci/mmol (44400-96200 GBq; end of synthesis).

**Toxicity Evaluation**

Normal male athymic nu/nu mice (5-6-week-old, Charles River, 490) were divided into three groups: CAN saline control, CAN-1, and CAN-10. Both right and left SMGs of mice in all groups were cannulated. Both SMG glands of mice in the CAN saline control group were infused with 50 µL of sterile saline; SMGs of mice in CAN-1 and CAN-10 were infused with 50 µL of 1 nmoles and 10 nmoles of DCFPyL, respectively. 1 week prior to performing cannulation, baseline saliva was collected from mice in all the groups, followed by the collection at 1-month and 2-month time-points. Groups of mice were monitored for a duration of 1 or 2 months for acute and chronic toxicity. Mice were weighed weekly and the end of the study periods. Mice were euthanized, and blood samples were collected to examine functional biomarkers such as BUN, blood albumin, ALT, AST, creatinine, amylase, blood bilirubin for kidney and liver functions. In the 2-month toxicity group, kidney, liver, and SMGs were removed and fixed in formalin; H&E staining on 5 µm formalin-fixed paraffin embedded sections was performed to evaluate histopathological differences in these organs between saline control, CAN-10, and CAN-1 groups. Histopathological examination was performed by a board-certified oral and maxillofacial pathologist.

**SUPPLEMENTARY FIGURES**

**
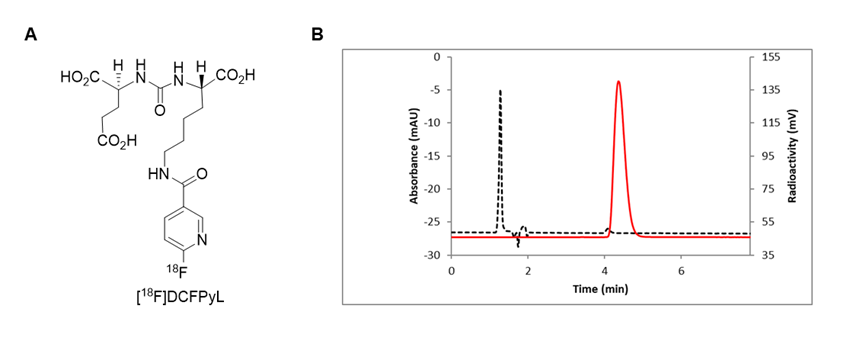
**

**SI Figure 1.** A: Structure of [^18^F]DCFPyL. B. HPLC analysis of [^18^F]DCFPyL (HPLC conditions: Agilent Eclipse plus C18 column (4.6 × 150 mm, 3.5 µm), mobile phase: 10% acetonitrile in 0.1 M ammonium formate, with a flow rate of 1.0 mL/min. Solid line: in-line radio detector; dotted line: UV detector at 254 nm.


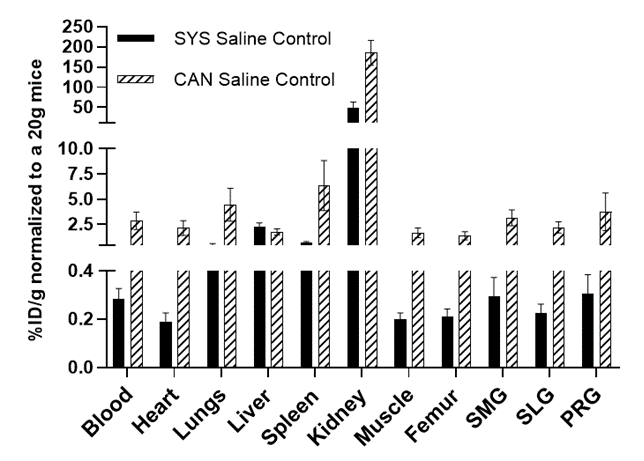


**SI Figure 2.** Biodistribution of [^18^F]DCFPyL in healthy mice at 1 h time-point. Mice in SYS saline control group were only injected (i.v.) with [^18^F]DCFPyL whereas mice in CAN saline control group were anesthetized and submandibular glands (SMG) were cannulated and infused with saline (50µl) followed by [^18^F]DCFPyL injection (i.v; 10 min after saline infusion). Submandibular gland:SMG, Sublingual gland:SLG, Parotid gland:PRG. Each bar represents mean %ID/g normalized to 20g ± SD; n=5 for each group.

**
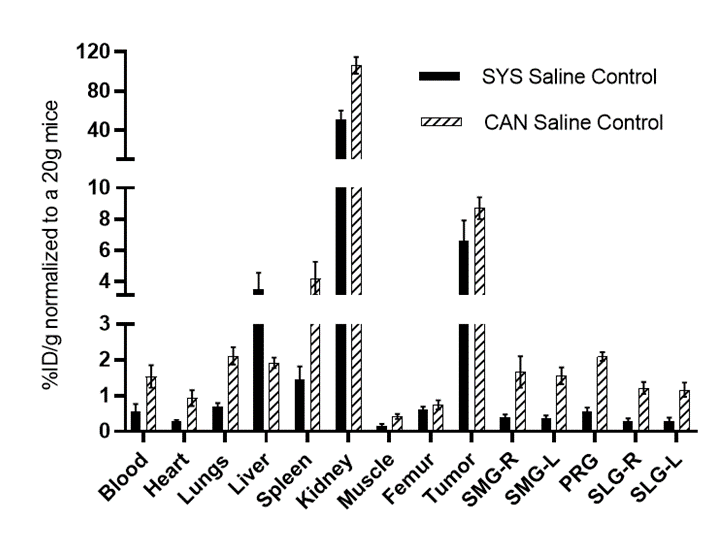
**

**SI Figure 3.** Biodistribution of [^18^F]DCFPyL in 22RV1 tumor bearing mice at 1 h. Mice in SYS saline control were only injected with [^18^F]DCFPyL whereas mice in CAN saline control group were anesthetized and submandibular glands (SMG) were cannulated and infused with saline followed by intravenous injection of [^18^F]DCFPyL (10 min after saline infusion). Submandibular gland:SMG, Sublingual gland:SLG, Parotid gland:PRG. R and L represents right and left. Each bar represents mean %ID/g normalized to 20g ± SD; n=5 for each group).


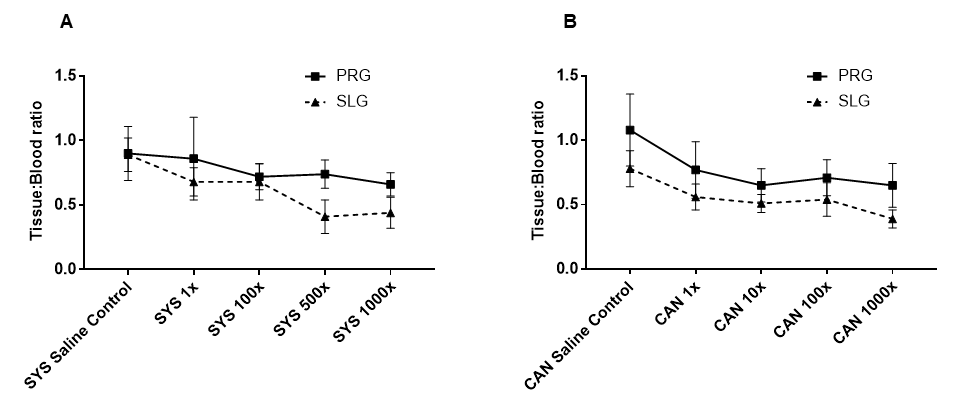


**SI Figure 4.** A: Tissue:Blood ratios of [^18^F]DCFPyL in parotid gland (PRG) and sublingual gland (SLG) at 1 h post-injection. Mice in SYS saline control group were injected with [^18^F]DCFPyL whereas mice in the blocking group (SYS 1x, SYS 100x, SYS 500x, SYS 1000x) were injected with [^18^F]DCFPyL + excess of DCFPyL. B: Tissue:Blood ratios of [^18^F]DCFPyL in PRG and SLG at 1 h post-injection. submandibular glands of mice were cannulated and infused with 50 µl of either saline (CAN saline control) or excess of DCFPyL (CAN 1x, CAN 10x, CAN 100x, CAN 1000x). 10 min after submandibular gland infusion mice were injected with [^18^F]DCFPyL. Each value in the graphs represents mean Tissue:Blood ratios ± SD, n=5-6 for each group. 1x, 10x,100x, 500x, and 1000x represents respective fold-excess doses of DCFPyL.


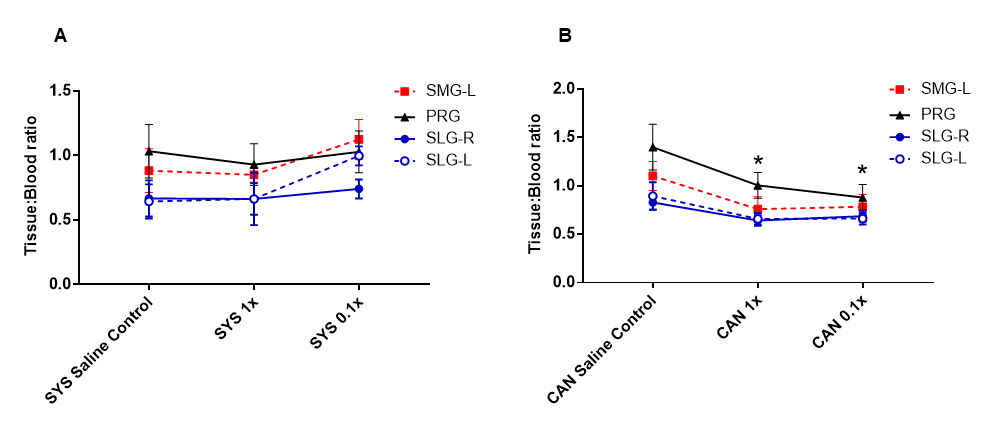


**SI Figure 5.** A: [^18^F]DCFPyL Tissue:Blood ratios of left submandibular gland (SMG-L), parotid gland (PRG), right and left sublingual gland (SLG-R, SLG-L) in mice bearing 22RV1 prostate cancer tumor. Mice in SYS saline control (SYS CTRL) group were injected with [^18^F]DCFPyL whereas mice in SYS 1x, SYS 0.1x were injected with [^18^F]DCFPyL + either 1 or 0.1-fold excess of DCFPyL. B: [^18^F]DCFPyL Tissue:Blood ratios of SMG-R, PRG, SLG-R, and SLG-L in mice bearing 22RV1 prostate cancer tumor. SMG glands were cannulated and infused with 50 µl of either saline (CAN saline control) or 1 or 0.1-fold excess of DCFPyL (CAN 1x, CAN 0.1x). 10 min after infusion of DCFPyL in the gland, mice were intravenously injected with [^18^F]DCFPyL. Each bar represents mean tissue:blood ratios ± SD, n=5-7 for each group.


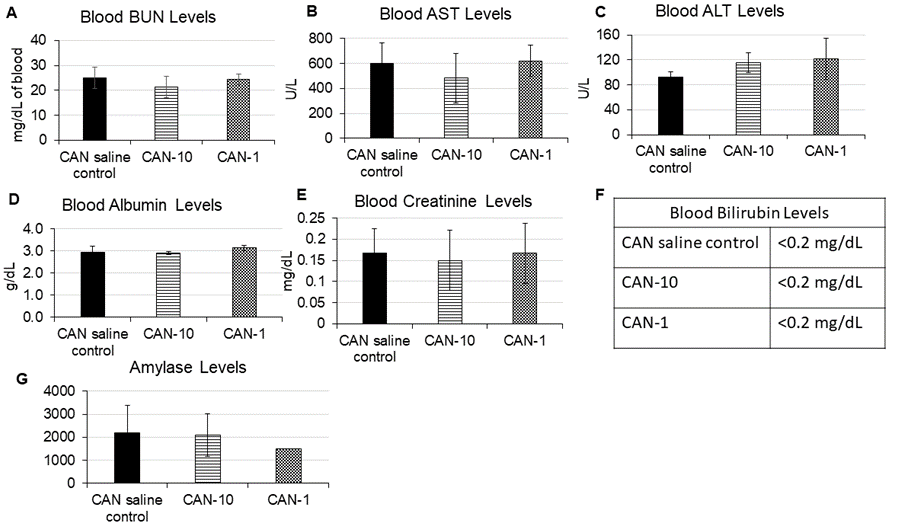


**SI Figure 6.** Blood BUN, AST, ALT, albumin, creatinine, bilirubin, and saliva amylase levels after 2 months post administration of either saline, 10 nmoles (CAN-10) or 1 nmoles (CAN-1) of unlabeled DCFPyL in submandibular glands of mice via cannulation. Each value represents mean value ± SD, n=4-6 for each group.
